# Tetracycline Molecularly Imprinted Fluorescent Sensor Based on Tomato Stalk-Derived Carbon Dots

**DOI:** 10.3390/s25226993

**Published:** 2025-11-15

**Authors:** Xuejing Wang, Jing Wang, Guanya Ji, Yihua Zhu, Jun Shi, Mengge Zhang, Chengshun Tang, Hongwei Duan, Xiuxiu Dong, Oluwafunmilola Ola, Qian Liu, Qijian Niu

**Affiliations:** 1 Key Laboratory of Modern Agricultural Equipment and Technology, School of Agricultural Engineering, Jiangsu University, Zhenjiang 212013, China; 2 College of Mechanical and Electrical Engineering, Shihezi University, Shihezi 832000, China; 3 Advanced Materials Research Group, University of Nottingham, Nottingham NG7 2RD, UK

**Keywords:** tomato straws, carbon dots, tetracycline, molecularly imprinted polymers, fluorescence sensor

## Abstract

**Highlights:**

**What are the main findings?**

- Novel carbon dots with excellent fluorescence were prepared from agricultural waste tomato straw.
- A fluorescence sensor made of CDs@SiO_2_-MIPs was constructed via molecularly imprinted SiO_2_-confined carbon dots.

**What are the implications of the main findings?**

- The developed sensor exhibited high selectivity, a wide linear range, and a low detection limit for TC detection.

**Abstract:**

In this work, novel biomass-derived carbon dots (CDs) with superior fluorescent properties were prepared from tomato straws. A selective, eco-friendly tetracycline (TC) sensor was fabricated by immobilizing a SiO_2_ molecularly imprinted polymer (MIP) layer onto CDs, forming a CDs@SiO_2_-MIP composite. This sensor combined highly selective adsorption properties with the sensitivity of fluorescence detection, with the sensing mechanism stemming from the off-fluorescent signal after molecular imprinting specifically recognizing the target substance. Under optimal conditions, the sensor exhibited a linear response to TC concentrations ranging from 1.00 × 10^−7^ to 5.00 × 10^−4^ mol/L, with fluorescence intensity decreasing as concentration increased. The detection limit of TC was 9.33 × 10^−8^ mol/L. This work provides novel biomass-derived CDs and a simple molecularly imprinted fluorescence sensing method for the detection of environmental organic pollutants.

## 1. Introduction

Tetracycline (TC) antibiotics, a class of broad-spectrum antimicrobial agents, find extensive application in both the medical and veterinary sectors. They are primarily utilized for treating bacterial infections and as feed supplements, leveraging their advantages of low cost and broad antibacterial activity [1,2,3]. However, excessive and continuous use of TC antibiotics has led to their residue in animal-derived foods, such as milk, meat, and eggs, as well as pollution of the water environment [4]. Upon entry into the human body via bioaccumulation, they can give rise to various adverse health effects, notably allergic reactions, hepatotoxicity, and gastrointestinal disturbance [5]. Currently, traditional methods including high-performance liquid chromatography (LC-ESI-MS-MS) [6], liquid chromatography–mass spectrometry (LC-MS) [7], and enzyme-linked immunosorbent assay (ELISA) [8] are widely employed for the detection of TC residues. However, in recent years, more cost-effective, convenient, and rapid detection methods compared to traditional approaches have been developed, including electrochemical, photoelectrochemical, colorimetric, and fluorescence methods. Among them, the fluorescence method has emerged as a promising approach in the field of analysis due to its advantages of high sensitivity, good accuracy, fast response speed, and low cost.

It is reported that there are various fluorescent materials that can be used to detect TC, including quantum dots [9,10,11], nanoclusters [12,13], metal–organic frameworks (MOFs) [14,15,16,17], and covalent organic frameworks (COFs) [18,19]. Among them, carbon dots (CDs), as a new type of luminescent nanomaterial, have attracted much attention because of their superior fluorescence characteristics, good solubility, and hypotoxicity [20,21]. In 2014, Yan et al. [22] reported a green strategy for preparing CDs by employing a biomass small molecule, citric acid, as the carbon precursor. Being natural, abundant, and renewable, biomass is an ideal carbon source for CD preparation, benefiting from its high carbon content and aromatic structure in components, which is conducive to the formation of conjugated carbon cores (sp^2^/sp^3^ hybridized), and the presence of heteroatoms such as N, S, and P, as well as rich functional groups such as -OH, -COOH, and -NH_2_ [23]. So far, a lot of biomass has been used to prepare carbon-based TC fluorescence sensors, such as rice residue [24], R. graveolens leaves [25], green jujube [26], passion fruit peels [27], Curcuma amada [28], and Ophiopogon japonicus f. nanus [29]. In all of these works, achieved good analytical performance was achieved. Nevertheless, the methodology for TC detection using CDs derived from tomato stalks remains to be investigated. Based on production volume, tomato stalks are the fourth most significant agricultural waste, after rice, wheat, and corn residues [30]. The presence of relatively high levels of pesticide and herbicide residues makes these vegetable residues ill-suited for use as silage [31,32]. Consequently, these vegetable residues are often discarded in landfills or incinerated on-site, leading to significant environmental pollution and resource waste [33]. Therefore, the construction of TC fluorescence sensors based on CDs derived from tomato straw not only effectively solves the problem of resource utilization of tomato straw waste in facilities but also realizes the effective detection of agricultural environmental pollutants, which has dual significance. However, the selectivity of fluorescence sensors based on pure biomass CDs requires further enhancement. The development of molecularly imprinted polymers (MIPs), inspired by the antigen–antibody principle, has established their potential for use as recognition units in sensor platforms, and they have become an ideal choice for improving the selectivity of fluorescence detection [34,35,36,37,38]. Their advantages include excellent physical and chemical stability, simple preparation, and low cost [39], and they can also withstand high temperatures, high pressures, acids, alkalis, and organic solvents [40]. Notably, silica (SiO_2_) possesses unique structural properties as an imprinting matrix [41]. Its surface is rich in hydroxyl groups (–OH), allowing for the convenient introduction of active groups such as amino groups (–NH_2_) via a silane coupling agent (3-aminopropyltriethoxysilane, APTES). The amino groups can serve as “imprinting sites” for the molecularly imprinted polymerization reaction, providing binding sites for the subsequent cross-linking polymerization of molecularly imprinted polymers (MIPs) [42]. Meanwhile, SiO_2_ exhibits excellent light transmittance, which prevents shielding or absorption of the fluorescence signal from carbon dots (CDs) [43]. Additionally, SiO_2_ is non-toxic and has good biocompatibility, eliminating the risk of secondary contamination to the test samples and meeting the requirements for “green sensing materials”. Therefore, encapsulating CDs with SiO_2_-MIPs not only enhances the sensitivity of fluorescence detection for tetracycline (TC) but also offers the advantages of low cost and environmental friendliness.

**Scheme 1:**
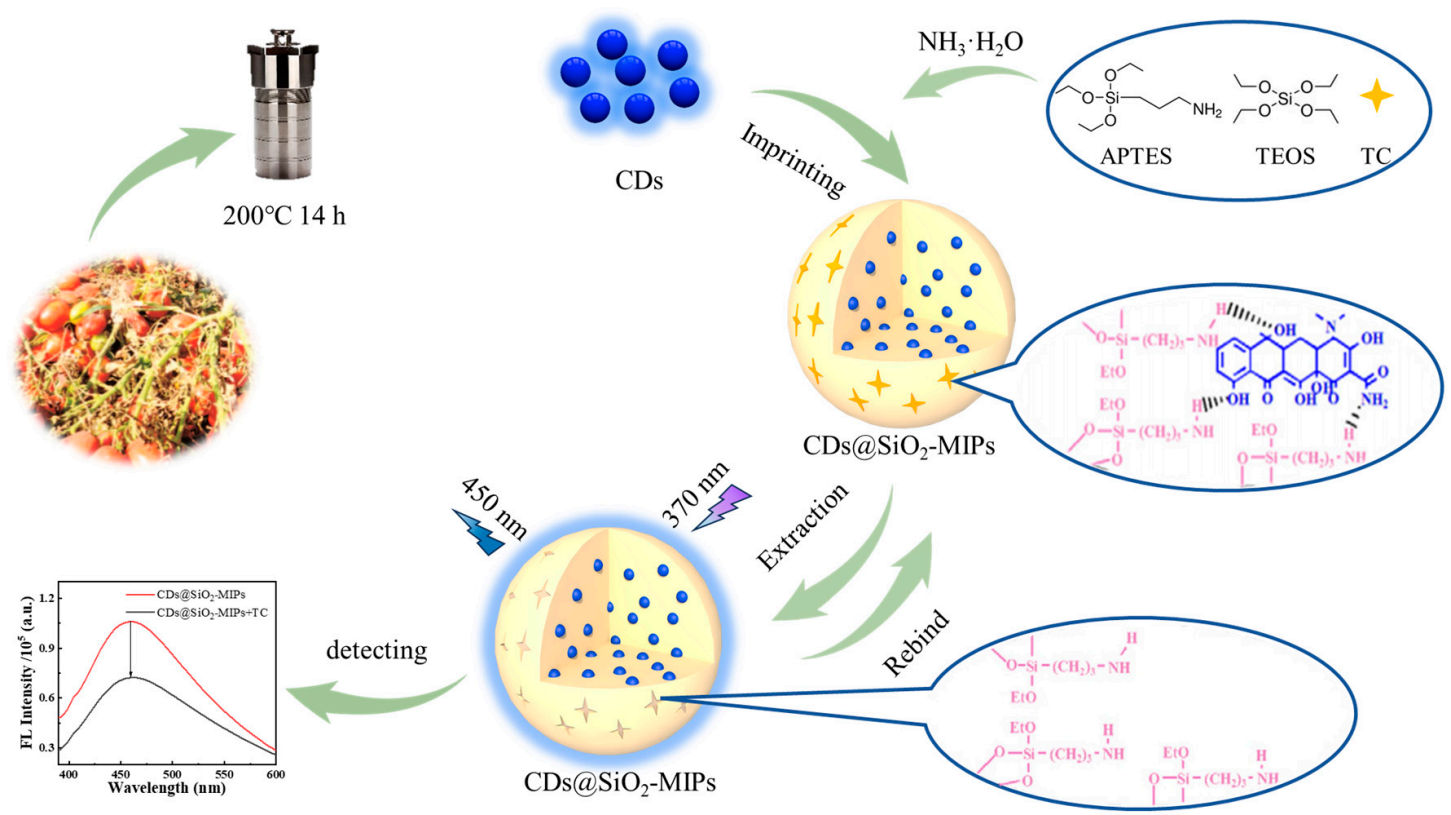
Schematic illustration of the preparation and detection process of CDs@SiO_2_-MIPs.

In this research, eco-friendly CDs were synthesized from tomato stalks and subsequently encapsulated into SiO_2_-MIPs via a sol–gel approach. The fluorescence of the CDs@SiO_2_-MIP sensor exhibited concentration-dependent quenching upon interaction with the TC molecule, which was attributed to the inner filter effect (IEF) and static quenching mechanism between TC and CDs@SiO_2_-MIPs (Scheme 1). The experimental results demonstrated that the CDs@SiO_2_-MIP sensor exhibited high selectivity, a wide linear range, and a low detection limit for TC. This study employed an eco-friendly, cost-effective, and simple approach to synthesize carbon dots (CDs) from renewable plant-based resources, while exploring their application in tetracycline (TC) detection. This work not only holds importance for researching CDs derived from natural products but also provides new insights into TC monitoring.

## 2. Materials and Methods

### 2.1. Materials

Tetracycline (TC), chlortetracycline (CTC), and sulfamethazine (SDM) were obtained from Macklin Biochemical Co., Ltd. (Shanghai, China). Oxytetracycline (OTC) and enrofloxacin (ENR) were obtained from InnoChem Science & Technology Co., Ltd. (Beijing, China). Tetraethoxysilane (TEOS) and 3-aminopropyl-triethoxysilane (APTES) were obtained from Sigma-Aldrich Trading Co., Ltd. (Shanghai, China). All other reagents, including ammonia solution (25%), acetic acid, methanol, and ethanol (analytical grade), were sourced from Sinopharm Chemical Reagent Co., Ltd. (Shanghai, China) and used as received. All experiments utilized Millipore Milli-Q ultrapure water (18.2 MΩ·cm).

### 2.2. Apparatus

Transmission electron microscopy (TEM) characterization was carried out using a Hitachi HT7800 high-contrast transmission electron microscope (Hitachi, Tokyo, Japan). For scanning electron microscopy (SEM) observations, a Hitachi Regulus-8100 field-emission scanning electron microscope (Hitachi, Tokyo, Japan) was employed. X-ray diffraction (XRD) patterns were acquired using a Bruker-D8 Advance diffractometer (Bruker, Karlsruhe, Germany). For X-ray photoelectron spectroscopy (XPS) analysis, an AXIS SUPRA spectrometer (Shimadzu, Kyoto, Japan) was employed. Ultraviolet–visible (UV-vis) spectra were recorded with a UV-3600 Plus UV-vis spectrophotometer (Shimadzu, Kyoto, Japan). Meanwhile, both fluorescence (FL) spectra and FL lifetime measurements were conducted using an FS5 fluorescence spectrophotometer (Edinburgh Instruments, Scotland, UK).

### 2.3. Preparation of CDs

The preparation of CDs was based on a literature method, which was adapted slightly for this work [44]. Briefly, fresh tomato stalks were collected from the Greenhouse located in Jiangsu University. The tomato stalks were first subjected to a cleaning and drying procedure, which involved rinsing with tap water and distilled water, followed by drying at 60 °C for 12 h in an incubator. The dried tomato stalks were manually crushed into a powder. Subsequently, 1.5 g of this powder was dissolved in 30 mL of ultrapure water and sonicated for 20 min to facilitate dispersion. Subsequently, the mixture was sealed in a 50 mL Teflon-lined autoclave and treated under hydrothermal conditions at 200 °C for a duration of 14 h. Finally, after cooling naturally to room temperature, the solution was centrifuged and filtered. The obtained dark brown liquid was stored at 4 °C for future use.

### 2.4. Preparation of CDs@SiO_2_-MIPs

In a typical procedure, 2 mL of CD solution and 10 mL of ethanol were added to a flask [45]. Following the addition of 80 µL of APTES, the mixture was stirred vigorously for 2 h to facilitate the self-assembly of APTES onto the CDs. Template TC (10 mg) was dissolved in ultrapure water (10 mL) and introduced into the aforementioned solution. After 15 min of stirring, 100 µL of ammonia hydroxide (25%) was added. Subsequently, a mixture of 100 µL TEOS and 10 mL ethanol was added dropwise under stirring. After being stirred at room temperature for 20 h, the resulting CDs@SiO_2_-MIPs were collected via centrifugation and purified by means of thorough washing with a methanol/acetic acid solution (95:5, *v*/*v*). To ensure complete removal of the TC template, the supernatant was checked by means of UV-vis spectroscopy following each wash. The washing process, typically repeated three times, was continued until no TC residue could be detected. The non-imprinted particles (CDs@SiO_2_-MIPs) were prepared using an identical procedure, with the only difference being that no template TC was added.

### 2.5. Fluorescent Sensing of TC

A fluorescent suspension of CDs@SiO_2_-MIPs (5 mg/mL) was prepared by ultrasonically dispersing 50 mg of the dried powder in 10 mL of ultrapure water. For detection, 500 μL of this suspension was mixed with 500 μL of a TC standard solution at varying concentrations. Subsequently, 800 μL of the mixture was transferred to a cuvette for fluorescence measurement. The spectrum was recorded from 380 to 600 nm upon excitation at 370 nm. The fluorescence emission signal at 450 nm was quantified. All data points reported represent the mean of three independent replicate measurements.

## 3. Results and Discussion

### 3.1. Characterization of CDs

**Figure 1:**
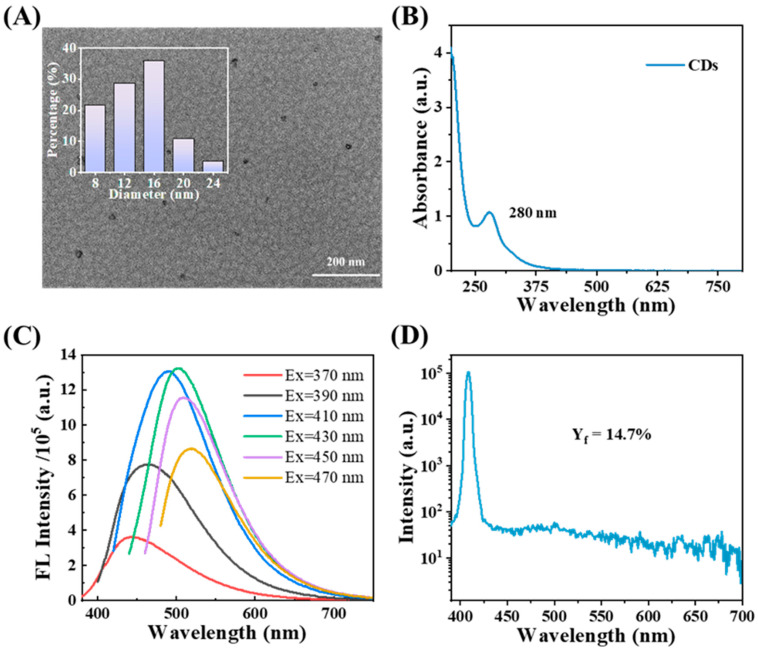
(**A**) TEM images of CDs; (**B**) UV–vis absorption spectra of CDs; (**C**) fluorescence spectra of CDs (excitation wavelength from 370 nm to 470 nm); (**D**) the FL quantum yield of CDs.

The morphology and particle size of biomass-derived CDs were characterized by means of TEM (Figure 1A). The CDs were spherical and relatively monodisperse, with average diameters of approximately 14.20 nm [44]. The optical properties of the as-prepared CDs were characterized by acquiring their UV-vis absorption and fluorescence spectra. As shown in Figure 1B, the CDs exhibited a strong absorption peak at 200 nm and a weaker one at 280 nm. The fluorescence spectra under excitations ranging from 370 to 470 nm are presented in Figure 1C. It became evident that the emission properties of the CDs were strongly influenced by the excitation wavelength. The optimal fluorescence intensity was achieved with excitation at 430 nm, yielding an emission peak at 500 nm. Shifting the excitation wavelength beyond 430 nm resulted in a red shift of the emission peak and a concurrent reduction in intensity. The quantum yield (QY) of the material, which is a critical parameter for assessing fluorescent performance, was measured with an FS5 FL spectrophotometer (Figure 1D). The QY of the CD solution fabricated via the above-mentioned method was quantified as 14.7%. This is a relatively high QY in CD solution directly prepared from biomass [46,47,48].

### 3.2. Characterization of CDs@SiO_2_-MIPs

**Figure 2:**
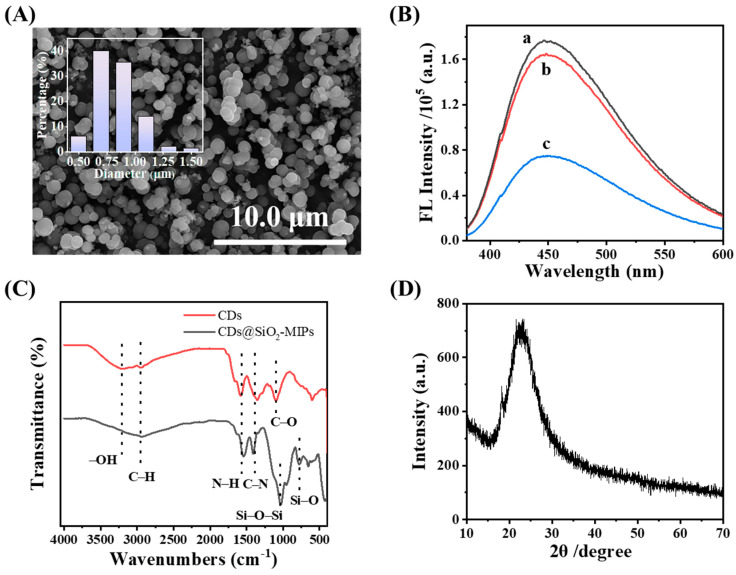
(**A**) SEM images of CDs@SiO_2_-MIPs; (**B**) fluorescence spectra of CDs@SiO_2_-NIPs (spectrum a) and CDs@SiO_2_-MIPs after (spectrum b) and before (spectrum c) the removal of TC; (**C**) FT-IR spectra of CDs, CDs@SiO_2_-MIPs; (**D**) XRD pattern of CDs@SiO_2_-MIPs.

The morphology of the CDs@SiO_2_-MIPs was characterized by means of TEM and SEM. Following this, the particle size was analyzed and the size distribution histogram was plotted using Nano Measurer 1.2. As shown in Figure 2A and Figure S1A,B, the synthesized CDs@SiO_2_-MIPs were regular spherical particles with an average particle size of approximately 0.83 μm. The size of CDs@SiO_2_-MIPs is significantly larger than that of CDs, and the imprinting layer of a single CDs@SiO_2_-MIP nanoparticle exhibited a rough morphology (Figure S1A), indicating that CDs have been completely encapsulated by the molecular imprinting layer. To monitor the effect of template extraction, synchronous fluorescence spectra (at 450 nm) were acquired for the CDs@SiO_2_-MIPs both before and after the removal process. The fluorescence quenching of the CDs@SiO_2_-MIPs (to 42.56% of the NIPs) and their subsequent recovery to 93.20% after elution (Figure 2B) can be attributed to the binding and subsequent removal of TC from the specific cavities, validating the successful creation of the molecularly imprinted sites. The result indicated that most of the TC molecules in the specific cavities of CDs@SiO_2_-MIPs could be washed out. Meanwhile, the fluorescence intensity of MIPs (spectrum b) is lower than that of NIPs (spectrum a), which is mainly caused by two factors: one is that the MIPs partially lose the luminescent signal of the CDs due to the formation of a relatively thick molecularly imprinted layer composed of functional monomers, as well as the combined effects of APTES, TC, and TEOS. In contrast, the NIPs lack an imprinted layer due to the absence of template molecules during polymerization, thus exhibiting a stronger fluorescence signal than the MIPs. The second one is that the fluorescence quenching can be attributed to non-radiative electron recombination induced by the specific alkylamine–carbonyl recognition on the quantum dots within the cavities [49]. Notably, a symmetric fluorescence emission peak appears at 450 nm when excited at 370 nm, and the optical characteristics of this peak match those of the CDs characterized above. Based on this correspondence, it can be inferred that the SiO_2_ coating on the surface of CDs exerts no restrictive effect on their photoluminescent properties [43]. Surprisingly, the phenomenon of blue shift in the maximum emission peak was observed after CDs were coated with SiO_2_-MIPs, as shown in Figure S2, which is presumably related to the polarization effect [50]. After the CDs are encapsulated, the SiO_2_-MIP layer forms a relatively rigid and low-polarity microenvironment around them. This change in the microenvironment restricts the intramolecular rotation and vibration of the CDs and reduces the non-radiative transition probability of the excited-state molecules of the CDs. To ensure the sensitivity and specificity of the sensing system, we re-optimized the excitation and emission wavelengths for the CDs@SiO_2_-MIP composite. An excitation wavelength of 370 nm and an emission wavelength of 450 nm were ultimately selected.

The FT-IR spectra of both the CDs and the CDs@SiO_2_-MIPs were characterized and compared, as presented in Figure 2C. The FT-IR spectrum of the CDs exhibited characteristic absorption bands corresponding to their functional groups. These include the presence of a C–H stretching vibration, as evidenced by the absorption band at 2949 cm^−1^, the broad adsorption of –OH groups between 3000 cm^−1^ and 3500 cm^−1^, the weak shoulder at around 1657–1750 cm^−1^ is assigned to –COOH groups, the band at 1578 cm^−1^ indicating the bending vibration of N–H, the band at 1351 cm^−1^ may originate from C–N stretching vibrations, while the band at 1093 cm^−1^ corresponds to C–O vibrations [51]. The FT-IR spectrum of the CDs@SiO_2_-MIPs showed strong, broad peaks at 956 cm^−1^ and 1033 cm^−1^, corresponding to Si–O–C and Si–O–Si asymmetric stretching, respectively. These characteristic peaks were absent in the spectrum of the CDs alone. The presence of peaks at approximately 778 cm^−1^ and 435 cm^−1^, indicative of Si–O vibrations, demonstrates that TEOS participated effectively in the synthesis process, forming a highly cross-linked network. All of the above spectral features collectively demonstrate the successful grafting of the imprinted polymers onto the CDs, formed via the sol–gel condensation of APTES and TEOS. Furthermore, the imprinting process does not alter the essential functional groups of the CDs or compromise their subsequent detection function.

As shown in Figure 2D, the XRD pattern for the CDs@SiO_2_-MIPs is characterized by a broad diffraction peak at approximately 2θ = 24°, corresponding to the C (002) graphitic plane. The XRD peak shows the amorphous nature of CDs@SiO_2_-MIPs.

**Figure 3:**
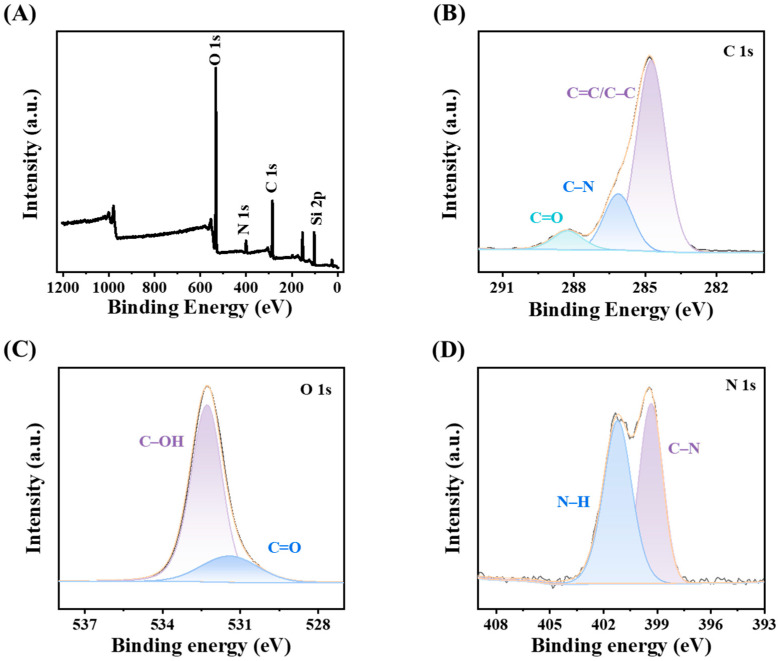
(**A**) XPS survey scan spectrum; The high-resolution spectrum of C 1s (**B**); O 1s (**C**); and N 1s (**D**).

The CDs@SiO_2_-MIPs were analyzed by means of XPS to determine their surface elemental composition, content, and chemical bonding states. In Figure 3A, the XPS survey spectrum shows four noticeable peaks with the binding energies of 285.2, 400.1, 531.8, and 102.9 eV that correspond to the C 1s, N 1s, O 1s, and Si 2p signals, respectively [52]. The high-resolution XPS spectra further elucidate the surface chemistry of the CDs@SiO_2_-MIPs. The C 1s spectrum (Figure 3B) was deconvoluted into three components at 284.8, 286.1, and 288.3 eV, assigned to C–C/C=C, C–N, and C=O bonds, respectively. Similarly, the O 1s spectrum (Figure 3C) showed two peaks at 531.4 eV (C=O) and 532.3 eV (C–OH), while the N 1s spectrum (Figure 3D) exhibited peaks at 399.3 eV (C–N) and 401.2 eV (N–H) [53]. These findings are fully consistent with the FT-IR results and further confirm that the CDs@SiO_2_-MIPs were generated successfully.

### 3.3. Optimization of the Sensor

Prior to measurements, a systematic optimization of the paramount factors affecting sensor performance was conducted to ensure ideal operating conditions. The mass ratio between the CDs and the functional monomer (APTES) was systematically optimized to maximize the fluorescence response of the sensing system. In Figure S3A, the optimal ratio of CDs to functional monomer (APTES) was 50:2, when the fluorescence intensity is maximum. Subsequent to molecularly imprinted polymerization, the successful elution of immobilized template molecules not only aids in the development of specific binding cavities and recognition sites but also stops the leakage of template molecules in the re-binding process; such leakage could result in false-positive readings. Therefore, the effects of different elution times on the elution efficiency of CDs@SiO_2_-MIPs were compared. As shown in Figure S3B, the fluorescence quenching efficiency reached its optimum at an elution time of 30 min. The decrease in quenching efficiency with prolonged time may be attributed to the degradation of imprinted cavities. Therefore, 30 min was selected as the optimal elution duration. The effect of incubation time was also investigated. Figure S3C shows that the fluorescence quenching efficiency of TC toward CDs@SiO_2_-MIPs increased rapidly within 0–20 min and remained constant after 20 min, confirming that the binding between TC and the imprinted cavities of CDs@SiO_2_-MIPs attained an equilibrium state in 20 min. To ensure effective binding of the imprinted cavities with TC, 25 min was selected as the optimal incubation time. An excessive amount of CDs@SiO_2_-MIPs would result in reduced sensitivity to TC, while an insufficient amount may lead to a narrow linear range. Therefore, the amount of CDs@SiO_2_-MIPs was optimized. As shown in Figure S3D, the fluorescence quenching efficiency was able to reach its highest value at the amount of 2.5 mg/mL. The fluorescence quenching efficiency decreased with decreasing amounts of CDs@SiO_2_-MIPs, while too high an amount of CDs@SiO_2_-MIPs (2.5 mg/mL) could lead to the self-quenching of fluorescence. Therefore, 2.5 mg/mL of CDs@SiO_2_-MIPs was selected as the optimal concentration.

### 3.4. Fluorescence Detection of CDs@SiO_2_-MIPs Toward TC

**Figure 4:**
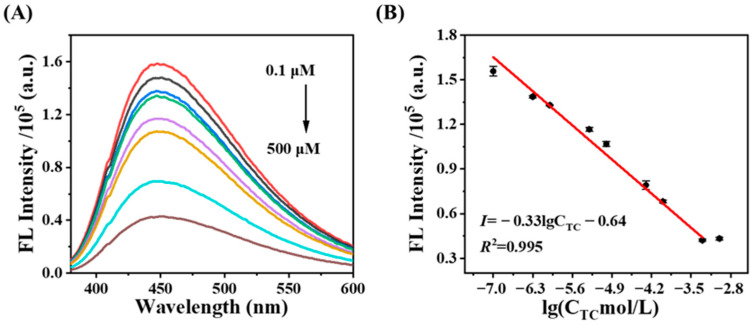
(**A**) Fluorescence responses of the CDs@SiO_2_-MIPs toward different concentrations of TC; (**B**) linear relationship between the logarithm of TC concentration and the fluorescence intensity (F_450_) of CDs@SiO_2_-MIPs.

Under the optimal conditions, the fluorescence signal of the CDs@SiO_2_-MIP sensor at 450 nm decreased with increasing concentrations of TC (Figure 4), which was linearly related to the logarithm of TC concentration, corresponding to the equation y = −0.33 lgC_TC_ − 0.64 (*R*^2^ = 0.995) (1 × 10^−7^~5 × 10^−4^ mol/L). Additionally, the limit of detection (LOD) for the CDs@SiO_2_-MIP system was calculated using the 3σ/K method. Here, σ denotes the standard deviation of blank sample measurements (with 10 parallel determinations, *n* = 10), and K represents the slope of the calibration curve derived from the concentration–response relationship. Thus, the LOD for CDs@SiO_2_-MIPs was 9.33 × 10^−8^ mol/L. Overall, the system offered outstanding sensitivity and a wide linear range.

### 3.5. Mechanism of CDs@SiO_2_-MIPs for TC Detection

**Figure 5:**
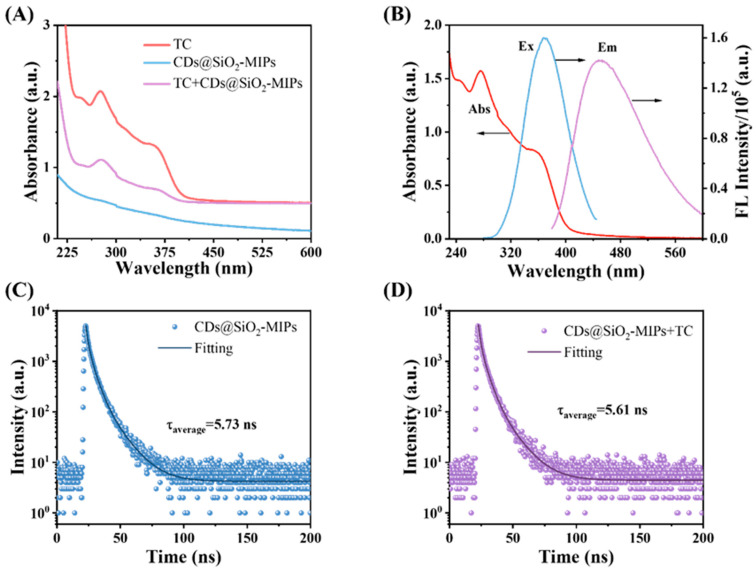
(**A**) UV–vis spectra of TC, CDs@SiO_2_-MIP nanocomposite with TC, CDs, and CDs@SiO_2_-MIP nanocomposite; (**B**) excitation and emission spectra of CDs@SiO_2_-MIPs and UV–vis absorption spectra of TC; fluorescence decay profile of the CDs@SiO_2_-MIPs in the absence (**C**) and presence (**D**) of TC.

The quenching mechanism was investigated through a series of designed experiments. Figure 5A depicts the UV-vis absorption peaks of TC, CDs@SiO_2_-MIPs, and CDs@SiO_2_-MIPs with TC. The UV-vis absorption peaks of TC are located at 276 nm and 357 nm, while CDs@SiO_2_-MIPs exhibit no significant absorption peaks. However, after mixing CDs@SiO_2_-MIPs with TC, the absorption peak of TC originally at 276 nm red-shifted to 282 nm. This indicated that the CDs@SiO_2_-MIPs nanoparticles interact with TC to form the ground state complex. To further illustrate the potential fluorescence quenching mechanism of the system, the quenching constant (*K_SV_*, m^−1^) was calculated using the following Stern–Volmer equation:

$$\frac{F_{0}}{F}=K_{sv}\left[Q\right]+1=k_{q}\tau_{0}\left[Q\right]+1$$

Among them, *F*_0_ and *F* represent the values of F_450_ in the CDs@SiO_2_-MIP system in the absence and presence of TC, respectively; [*Q*] is the concentration of TC (μM); *k_q_* is the quenching rate constant; and *τ*_0_ is the fluorescence lifetime in the absence of TC, which is 5.73 ns. As shown in Figure S4, the *K_SV_* value was calculated from the slope of the fitted curve (*K_SV_* = 7.10 × 10^3^ M^−1^). The *k_q_* value was obtained using the formula (*k_q_* = *K_SV_*/*τ*_0_), which is 1.24 × 10^12^ M^−1^s^−1^. When the *k_q_* value is greater than 2.0 × 10^10^ M^−1^s^−1^, it indicates that the quenching mechanism of the system involves static quenching [54]. In the temperature-dependent quenching experiment (Figure S4), the *K_SV_* of the system decreases sequentially: *K_SV_* = 7.10 × 10^3^ M^−1^ at 35 °C, 6.80 × 10^3^ M^−1^ at 45 °C, and 6.19 × 10^3^ M^−1^ at 55 °C. This negative temperature dependence confirms that the interaction between CDs@SiO_2_-MIPs and TC is a ground-state interaction, rather than a collisional quenching process [55].

In addition, as shown in Figure 5B, the significant spectral overlap between the ultraviolet absorption peak of TC at 357 nm and the excitation spectrum of the CDs@SiO_2_-MIPs suggests that fluorescence quenching likely occurs via the inner filter effect (IFE) and/or Förster resonance energy transfer (FRET). IFE is a non-radiative energy loss mechanism whereby the excitation or emission light is absorbed by other species in the system, resulting in a decrease in the measured fluorescence intensity [56], whereas FRET is a distance-dependent energy transfer phenomenon from the excited-state donor (CDs@SiO_2_-MIPs) to the ground-state acceptor (the quencher) [35]. The fluorescence lifetime will shorten if quenching occurs via the non-radiative FRET pathway, but it will remain unchanged if the attenuation is due to the radiative IFE [57]. The fluorescence lifetime of the CDs@SiO_2_-MIPs was measured to elucidate the quenching mechanism. As shown in Figure 5C,D, the lifetimes before and after TC addition were 5.73 ns and 5.61 ns, respectively. In addition, as shown in Figure S5, the fluorescence lifetimes of the CDs@SiO_2_-MIPs system under different TC concentration gradients were also measured. The negligible change rules out FRET and confirms that the quenching is caused by the IFE [28]. When the excitation light was irradiated, the accumulated TC on the sensor surface acted as an inner filter, absorbing the excitation light and thereby attenuating the energy absorbed by the CDs, which resulted in significant fluorescence quenching.

### 3.6. Selectivity, Anti-Interference Ability, and Stability of the CDs@SiO_2_-MIP Sensor

**Figure 6:**
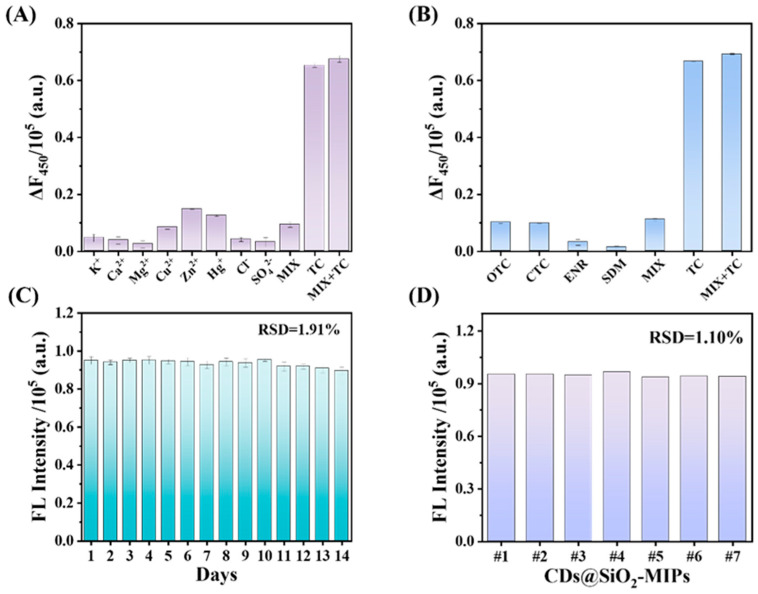
(**A**) Influence of coexisting common ions (250 μM); (**B**) quenching effects of synchronous fluorescence of CDs@SiO_2_-MIPs to OTC, CTC, ENR, SDM, and TC in water (each of the concentrations was 250 μM); (**C**) the stability of the fluorescence sensing system over time; (**D**) the precision of sensors constructed in different batches.

The sensing system was further evaluated for its selectivity, anti-interference capability, and stability. The selectivity was assessed by challenging the sensor with TC, other antibiotics (OTC, CTC, ENR, and SDM), and potential interfering ions (K^+^, Ca^2+^, Mg^2+^, Cu^2+^, Zn^2+^, Hg^+^, Cl^−^, and SO_4_^2−^), as shown in Figure 6A,B. The sensor caused pronounced fluorescence quenching with 25 μM TC, while even a tenfold higher concentration (250 μM) of competing antibiotics or ions induced negligible signal change. Despite the structural similarity of OTC and CTC to TC, their fluorescence responses were negligible even at 10-fold higher concentrations. As shown in Figure S6, the fluorescence quenching efficiencies of CDs by TC, OTC, and CTC were also measured. The results indicate that all three exhibit obvious fluorescence quenching phenomena. In the experiment of TC-induced fluorescence quenching of CDs@SiO_2_-NIPs, the response was relatively weak compared with that of CDs@SiO_2_-MIPs. This high specificity underscores the sensor’s exceptional selectivity for TC. Furthermore, the fluorescence signal remained stable when TC was detected in the presence of these interferents, confirming the sensor’s excellent anti-interference capability.

Furthermore, the CDs@SiO_2_-MIPs exhibited excellent stability, maintaining nearly constant performance for detecting 25 μM TC even after 14 days of storage at 4 °C (Figure 6C). To assess the batch-to-batch reproducibility, TC was detected using CDs@SiO_2_-MIPs synthesized in different batches, all of which yielded a consistent response (Figure 6D). The calculated RSD is 1.01%, indicating the high accuracy of the sensing system.

Optical stability is of paramount importance for fluorescent sensors, governing their practical reliability. In Figure S7, both CDs and CDs@SiO_2_-MIPs exhibited minimal fluorescence intensity variations when exposed to 365 nm UV irradiation, demonstrating relative standard deviations (RSDs) of merely 0.63% and 0.94% over 120 min, respectively [58]. These results confirm the remarkable photobleaching resistance of the developed sensor.

### 3.7. Comparison with MIP-Based Sensors for TC Detection

Benchmarked against previously reported MIP-based fluorescent sensors (Table S1), the proposed sensor demonstrates superior analytical performance, including a lower detection limit and a wider linear range, enhancing its applicability for TC monitoring in diverse matrices. Furthermore, many sensor preparation protocols reported in the literature involve the use of hazardous reagents, including volatile toxins [59,60] and strong acids [61] and bases [62], which pose risks to health and the environment. Additionally, these methods often suffer from cumbersome and complex processes [63,64]. The preparation process for CDs@SiO_2_-MIPs is straightforward and utilizes inexpensive, low-toxicity raw materials, better aligning with the principles of green chemistry.

## 4. Conclusions

In this work, we developed a novel sensor based on CDs@SiO_2_-MIP fluorescence quenching for the facile and effective determination of TC. Briefly, fluorescent CDs were synthesized from tomato stalks via a green hydrothermal method in the aqueous phase without additional chemicals. Subsequently, the CDs@SiO_2_-MIP sensor was engineered through sol–gel polymerization with the aim of significantly improving its sensitivity and selectivity. Based on the UV-Vis absorption spectra and fluorescence lifetime data, the quenching mechanism is attributed to a synergy between the IFE and static quenching. This study provides a green and simple method for the recycling and reuse of tomato stalks from an agricultural waste facility. Meanwhile, the functional carbon dots derived from tomato stalks can be used as a fluorescent sensing probe for the rapid and reliable investigation of environmental pollutants.

## Data Availability

Data are contained within the article or Supplementary Material. Any additional data concerning the work in this study are available from the corresponding author upon request.

## Supplementary Materials

The following supporting information can be downloaded at https://www.mdpi.com/article/10.3390/s25226993/s1. Figure S1: (A) SEM and (B) TEM images of CDs@SiO_2_-MIPs; Figure S2: The fluorescence spectra of CDs@SiO_2_-MIPs (excitation wavelength from 350 nm to 390 nm); Figure S3: (A) FL Intensity of CDs and APTES at different volume ratios. Effect of (B) elution time; (C) incubation time; (D) CDs@SiO_2_-MIPs concentration on CDs@SiO_2_-MIP detection performance; Figure S4: The temperature effect on the quenching process of CDs@SiO_2_-MIP complexes by TC; Figure S5: Fluorescence lifetime of the CDs@SiO_2_-MIP system at different TC concentrations; Figure S6: Quenching effects of synchronous fluorescence of CDs to OTC, CTC, and TC and CDs@SiO_2_-MIPs and CDs@SiO_2_-NIPs to TC; Figure S7: The fluorescence stability property of CDs (A) and CDs@SiO_2_-MIPs (B); Table S1: The comparison of the proposed method with other reported methods of TC fluorescence detection. References [26,27,65,66,67,68,69,70,71] are cited in the Supplementary Materials.
