# Supplementary material for: Tetracycline Molecularly Imprinted Fluorescent Sensor Based on Tomato Stalk-Derived Carbon Dots
Source: Sensors (Basel). 2025 Nov 15;25(22):6993. doi: 10.3390/s25226993 (PMC12656203; doi:10.3390/s25226993)
Supplement: Supplementary file 1 [file sensors-25-06993-s001.zip › sensors-3952688-supplementary.pdf]

Supplementary Materials

Tetracycline molecularly imprinted fluorescent sensor based on tomato stalk-derived carbon dots

Xuejing Wang [1], Jing Wang [1], Guanya Ji [1], Yihua Zhu [1], Jun Shi [1], Mengge Zhang [1], Chengshun Tang [1], Hongwei Duan [2], Xiuxiu Dong [1], Oluwafunmilola Ola [3], Qian Liu [1], Qijian Niu [1,*]

[1] Key Laboratory of Modern Agricultural Equipment and Technology, School of Agricultural Engineering, Jiangsu University, Zhenjiang, Jiangsu 212013, China

[2] College of Mechanical and Electrical Engineering, ShiHezi University, Shihezi, Xinjiang 832000, China.

[3] Advanced Materials Research Group, University of Nottingham, NG7 2RD, UK

* Corresponding author: Qijian Niu E-mail: niuqijian@ujs.edu.cn

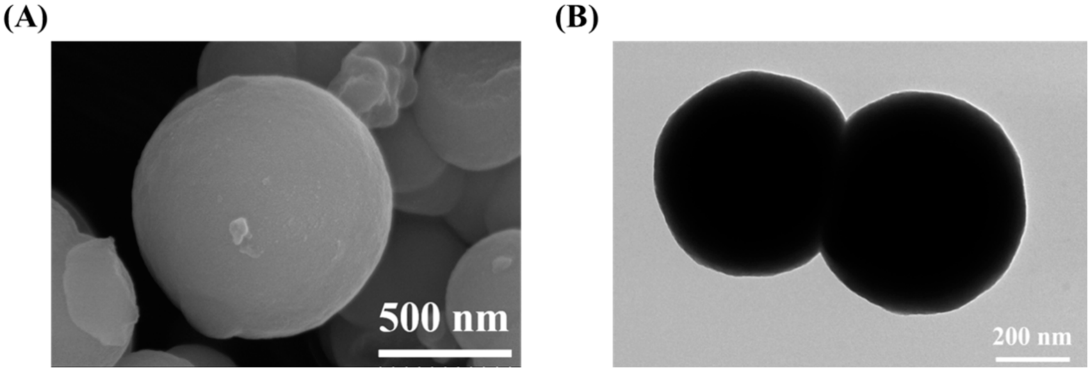

Figure S1. (A) SEM and (B) TEM images of CDs@SiO_2-MIPs.

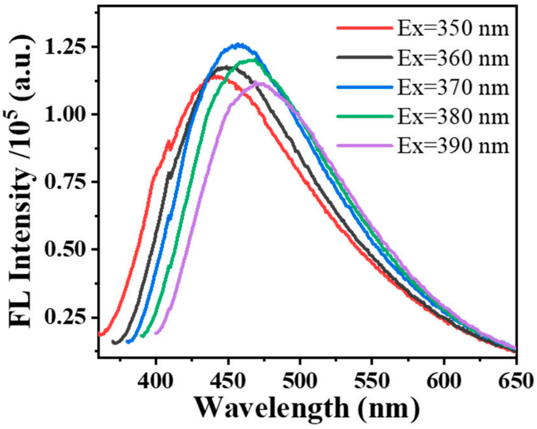

Figure S2. The fluorescence spectra of CDs@SiO_2-MIPs (excitation wavelength from 350 nm to 390 nm)

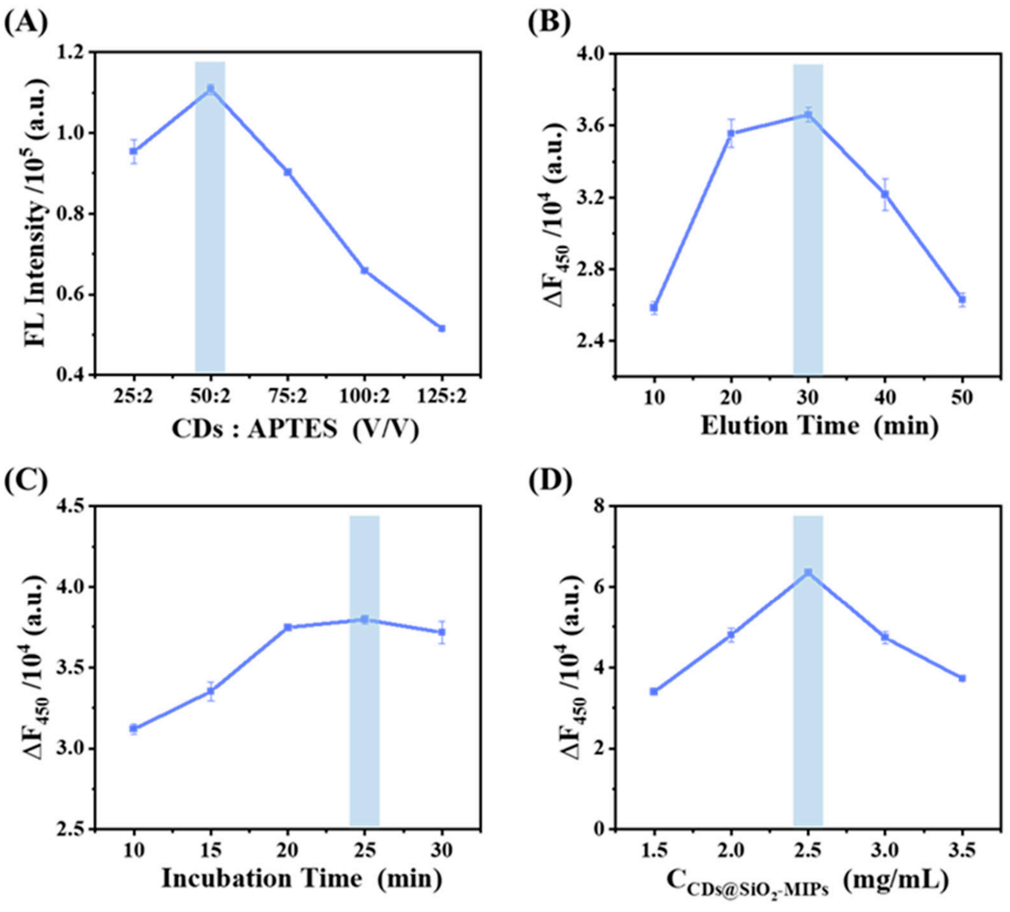

Figure S3. (A) FL Intensity of CDs and APTES at different volume ratios; Effect of (B) elution time; (C) incubation time; (D) CDs@SiO2-MIPs concentration on CDs@SiO2-MIPs detection performance.

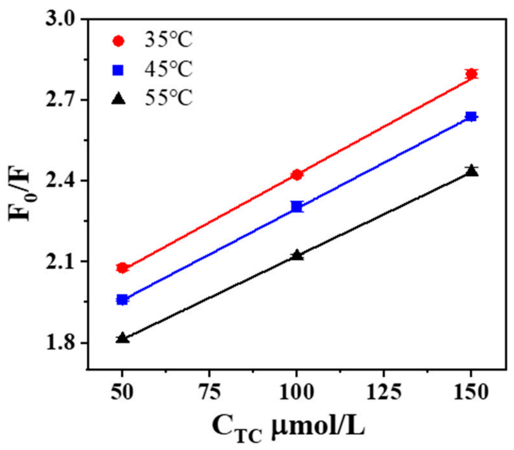

Figure S4. The temperature effect on the quenching process of CDs@SiO2-MIPs complexes by TC.

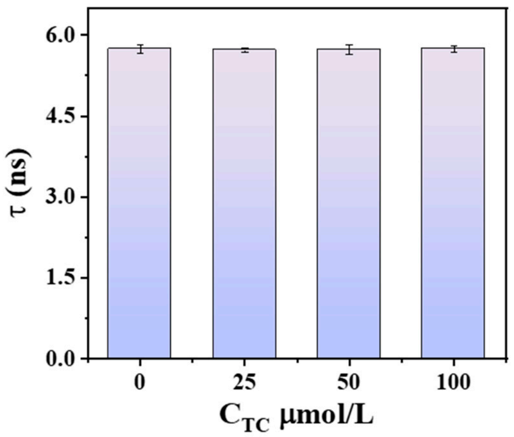

Figure S5. Fluorescence lifetime of the CDs@SiO_2-MIPs system at different TC concentrations.

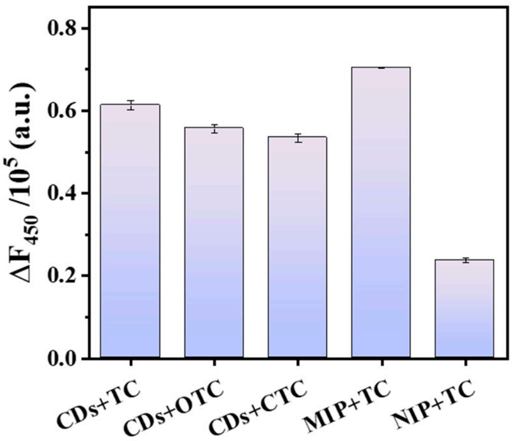

Figure S6. Quenching effects of synchronous fluorescence of CDs to OTC, CTC and TC, CDs@SiO_2-MIPs and CDs@SiO_2-NIPs to TC.

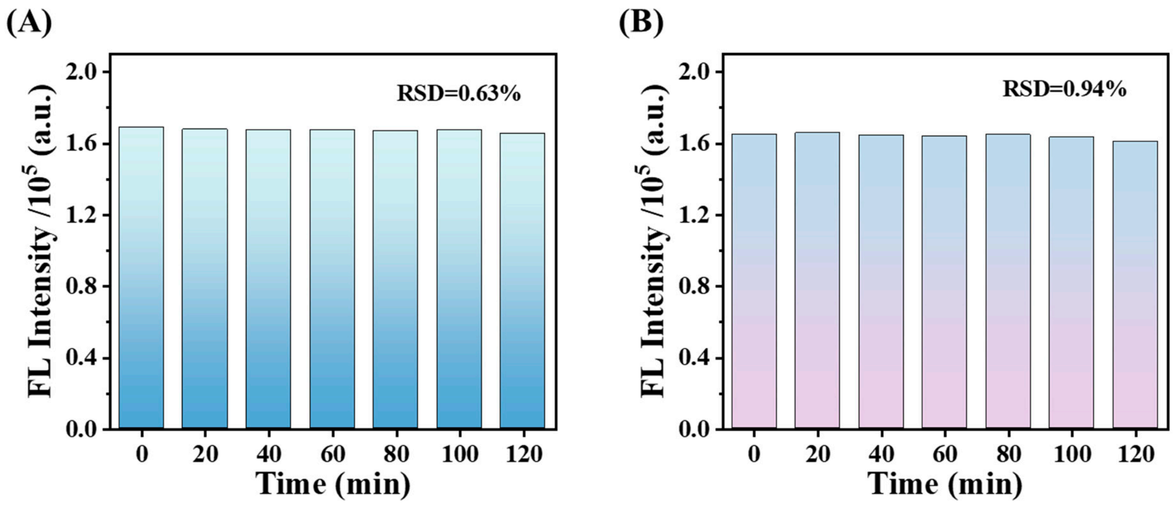

Figure S7. The fluorescence stability property of CDs (A) and CDs@SiO_2-MIPs (B).

Table S1 The comparison of the proposed method with other reported methods of TCs fluorescence detection.

Carbon source	Material	Target	Linear range (mol/L)	LOD (mol/L)	References
citric acid	S, N-CDs	TCs	$0.00–6.00\times10^{-5}$	2.50×10^{-7}	[1]
carrot juice	CDs	TC	$4.00\times10^{-6}–1.55\times10^{-5}$	1.33×10^{-6}	[2]
wild lemon leaves	LLCDs	TCs	$0.00–2.70\times10^{-5}$	4.20×10^{-7}	[3]
green jujube	B-CDs	TCs	$0.00–1.00\times10^{-7}$	6.10×10^{-8}	[4]
citric acid	Eu-CDs	TCs	$0.00–6.24\times10^{-4}$	1.58×10^{-8}	[5]
puffer fish skin	N-CDs	TCs	$5.00\times10^{-7}–5.00\times10^{-4}$	1.50×10^{-7}	[6]
citric acid	CDs-MIPs	TC	$1.13\times10^{-6}–6.75\times10^{-5}$	1.22×10^{-7}	[7]
passion fruit peel	B-CQDs@Eu/MIPs	TC	$2.50\times10^{-8}–2.00\times10^{-6}$	7.9×10^{-9}	[8]
citric acid	Mg, N-CDs@MIPs	TC	$1.13\times10^{-8}–2.25\times10^{-7}$	1.78×10^{-9}	[9]
**tomato stalks**	**CDs@SiO_2-MIPs**	**TC**	**$1.00\times10^{-7}–5.00\times10^{-4}$**	**9.33×10^{-8}**	**This work**

References

1. Xing, X.; Huang, L.; Zhao, S.; Xiao, J.; Lan, M., S,N-Doped carbon dots for tetracyclines sensing with a fluorometric spectral response. *Microchemical Journal* **2020,** 157, 105065.
2. Mohammadnejad, M.; Alekasir, R., Sensitive and rapid determination of tetracycline antibiotic by carrot juice-derived carbon dots as a fluorescent probe. *Luminescence* **2024,** 39, e4828.
3. Venugopalan, P.; Vidya, N., Microwave-assisted green synthesis of carbon dots derived from wild lemon (Citrus pennivesiculata) leaves as a fluorescent probe for tetracycline sensing in water. *Spectrochimica Acta Part A: Molecular and Biomolecular Spectroscopy* **2023,** 286, 122024.
4. Hu, J.; Liao, S.; Bai, Y.; Wu, S., Carbon dots derived from green jujube as chemosensor for tetracycline detection. *Journal of Environmental Chemical Engineering* **2024,** 12, 112595.
5. Fan, Y. J.; Wang, Z. G.; Su, M.; Liu, X. T.; Shen, S. G.; Dong, J. X., A dual-signal fluorescent colorimetric tetracyclines sensor based on multicolor carbon dots as probes and smartphone-assisted visual assay. *Analytica Chimica Acta* **2023,** 1247, 340843.
6. Yan, W.; Wang, X.; Gao, X.; Zhao, L., A smart fluorescent colorimetric dual-response sensing for the determination of tetracycline antibiotics. *Journal of Photochemistry and Photobiology A: Chemistry* **2024,** 447, 115217.
7. Wang, Q.; Wu, Y.; Bao, X.; Yang, M.; Liu, J.; Sun, K.; Li, Z.; Deng, G., Novel fluorescence sensor for the selective recognition of tetracycline based on molecularly imprinted polymer-capped N-doped carbon dots. *RSC Advances* **2022,** 12, 24778-24785.
8. Sun, X.; Jiang, M.; Chen, L.; Niu, N., Construction of ratiometric fluorescence MIPs probe for selective detection of tetracycline based on passion fruit peel carbon dots and europium. *Microchimica Acta* **2021,** 188, 297.
9. Hu, X.; Zhao, Y.; Dong, J.; Liu, C.; Qi, Y.; Fang, G.; Wang, S., A strong blue fluorescent nanoprobe based on Mg/N co-doped carbon dots coupled with molecularly imprinted polymer for ultrasensitive and highly selective detection of tetracycline in animal-derived foods. *Sensors and Actuators B: Chemical* **2021,** 338, 129809.
